# Evolution of a sustained health promotion programme exploring adolescent metabolic health in the Cook Islands

**DOI:** 10.1093/heapro/daaf069

**Published:** 2025-06-26

**Authors:** Jacquie L Bay, Tania John, Celeste Barrett-Watson, Karen Ngamata, Amy Renelle, Suzanne A Trask, Metua Bates, Mark H Vickers, Danielle Tungane Cochrane

**Affiliations:** Liggins Institute, Waipapa Taumata Rau The University of Auckland, Auckland 1142, New Zealand; Koi Tu: The Centre for Informed Futures, Waipapa Taumata Rau The University of Auckland, Auckland 1142, New Zealand; Publich Health, Te Marae Ora Cook Islands Ministry of Health, Rarotonga, Cook Islands; Maraurau o te Pae Api’i, Cook Islands Ministry of Education, Rarotonga, Cook Islands; Publich Health, Te Marae Ora Cook Islands Ministry of Health, Rarotonga, Cook Islands; Liggins Institute, Waipapa Taumata Rau The University of Auckland, Auckland 1142, New Zealand; Liggins Institute, Waipapa Taumata Rau The University of Auckland, Auckland 1142, New Zealand; Koi Tu: The Centre for Informed Futures, Waipapa Taumata Rau The University of Auckland, Auckland 1142, New Zealand; Publich Health, Te Marae Ora Cook Islands Ministry of Health, Rarotonga, Cook Islands; Liggins Institute, Waipapa Taumata Rau The University of Auckland, Auckland 1142, New Zealand; Liggins Institute, Waipapa Taumata Rau The University of Auckland, Auckland 1142, New Zealand; Maraurau o te Pae Api’i, Cook Islands Ministry of Education, Rarotonga, Cook Islands

**Keywords:** school-based health promotion, adolescent, Cook Islands, Pacific Island, noncommunicable disease, metabolic health, community-based participatory research, Tivaevae

## Abstract

The non-communicable disease (NCD) burden in the Cook Islands is severe; 62% of adults 18–69 years live with 3–5 risk factors. Understanding NCD complexity and developing evidence-based actions to mitigate this burden is crucial. This article reports on the evolution of a sustained health promotion programme contributing to understanding adolescent metabolic health in Rarotonga. Ora’anga Tūmanava (established 2013) is a transdisciplinary initiative engaging adolescents in exploring NCD-related challenges through curriculum-linked learning. Community-based participatory research integrated within the Tivaevae framework guides co-design. In 2013, inclusion of health measures within this initiative was considered but rejected. Feedback in 2015 identified that, after examining population-level NCD burdens, adolescents wanted greater access to personal health information. Consequently, inclusion of health measures was revisited and agreed for trial alongside learning about metabolic health within a Year 9 programme. Data from 2016 to 2019 and 2022 to 2023 (*n* = 783; 65% total cohort; median age 13.8 years) indicated overweight 22.1%; obesity 37.6%; waist-to-height ratio > 0.5, 39.1%; elevated blood cholesterol, 8.1%; and elevated blood glucose, 15.6%. Where all measures were available (*n* = 321), 27.7% of students were living with ≥ 3 risk factors. Feedback confirmed the programme met student expectations, contributed to school-based health promotion, offered an acceptable strategy for tracking metabolic health indicators and highlighting NCD risk factors in this age-group, and encouraged health-related discussions. Alignment with curriculum-based learning and evidence-sharing has ensured sustained school-level support. This study highlights how transdisciplinary partnerships built via culturally appropriate co-design can support educational and health promotion goals and simultaneously inform public health in small island communities.

Contribution to Health Promotion

- This research highlights the value of health-education partnerships to enable sector-specific goals without compromising practice-based values.
- It highlights the importance of incorporating cultural values into approaches to community-based participatory research.
- It emphasises listening to young people and creating opportunities for them to access and understand health data through curriculum-linked learning.
- It demonstrates that sustainability of school-based health promotion is supported when co-design recognises education perspectives and invests in partnership development.
- The findings highlight the NCD risk burden carried by the Cook Islands population and the value of school-based partnerships for non-communicable disease risk reduction in Pacific communities.

## INTRODUCTION

The Cook Islands (population 14 987) is a self-governing state in free association with New Zealand, comprising 15 small islands spread across a 2 000 000 km^2^ exclusive economic zone in the southern Pacific Ocean. Seventy percent of the population live on the island of Rarotonga. Like many Pacific Island nations, the Cook Islands face significant challenges associated with the prevalence of non-communicable diseases (NCD). The WHO STEPwise approach to NCD risk factor surveillance (STEPS) survey, which measures key behavioral and biological factors, is designed to monitor population-level NCD risk, particularly in low-resource settings (WHO 2005, Riley *et al*. 2016). The 2022 Cook Islands STEPS survey indicated that 99% of adults aged 18–69 live with at least one, and 62% with 3–5 NCD risk factors. This is evident from the high rates of overweight (18%) and obesity (75%). Less visible are the type 2 diabetes burden, managed by one in three adults, and high rates of premature NCD-related mortality (Te Marae Ora Cook Islands Ministry of Health 2023).

The context of Small Island Developing States (SIDS) magnifies the challenges associated with NCDs in the Pacific (Connell *et al*. 2020, Guell *et al*. 2024). Factors including geographic isolation, small size, limited economic and human capacity, difficulty in providing health services, and vulnerability to natural disasters interact within the system in which actions to address NCD-related challenges are situated. For over 25 years, the Cook Islands have prioritised understanding NCD complexity and developing evidence-based strategies to mitigate social and economic impacts. These strategies align with the socio-ecological health promotion approach in the Pacific Healthy Islands Framework, acknowledging the holistic, culturally embedded nature of well-being and the necessity for transdisciplinary approaches to health promotion (Nutbeam 1996, Galea *et al*. 2000). Alongside meeting the needs of people living with NCDs are interventions to reduce future NCD burden. One such initiative is the Pacific Science for Health Literacy Project (PSHLP), established in 2013 and renamed Ora’anga Tūmanava in 2024, marking its transition to a sustained national programme (Tu’akoi *et al*. 2024). This transdisciplinary partnership among education, health, science, and school communities aims to promote educational development and reduce NCD risk in adolescents and their future offspring (Bay *et al*. 2016, 2019, Bay 2017). The formation of this partnership between the Cook Islands Ministry of Education, Te Marae Ora Cook Islands Ministry of Health, and the University of Auckland was facilitated by the New Zealand Ministry of Foreign Affairs and Trade (MFAT; *Ora’anga Tūmanava: Our History* 2024). Uncharacteristically, MFAT did not propose a detailed agenda. Instead, recognising potential synergies, funding was allocated to facilitate meetings among prospective partners, enabling them to share narratives regarding approaches to health and education challenges and to explore opportunities for collaboration. Consequently, the partners developed a successful *Partnerships for International Development* proposal to foster critically engaged citizenship and contribute to a reduction in NCD risk factors in the long-term (Bay and McIntyre 2013). Thus, co-design was established at the inception of PSHLP, ensuring that the focus and approach were determined locally, thereby fostering the building of trust, shared understanding, and valuing each partner’s input from the outset (Winterbauer *et al*. 2016).

PSHLP/Ora’anga Tūmanava acknowledges the transgenerational nature of NCD risk (Watson 2024), the critical intervention period of adolescence (Hanson and Gluckman 2014, Patton *et al*. 2018, Reinehr 2018), and the complexity of NCDs in Pacific Island nations (Tu’akoi *et al*. 2018). While engaging adolescents, the initiative also seeks to enhance understanding in education, health, government, and the community about the significance of adolescence as a key intervention point.

The undisputed link between education, health, and well-being highlights the opportunity for schools to promote health and the need for collaboration between education and health sectors (Gray *et al*. 2022). Integrating health programmes into academic, pastoral, and environmental aspects of schools is essential (World Health Organization 2017, Bradley-Klug *et al*. 2019). Co-design that combines pedagogical expertise with health-related evidence ensures these programmes become part of regular operations, adding value and offering sustained impacts (Bay et al. 2016a). Engaging school leaders is crucial (Adams *et al*. 2023), as is incorporating adolescent voices in programme design (Gray *et al*. 2022).

In 2013, deliberations during the co-design of the PSHLP monitoring and evaluation plan considered the inclusion of measures associated with metabolic syndrome in the adolescent population. Measures of height, weight, waist circumference, blood pressure (BP), fasting blood glucose, and cholesterol would have been required (DeBoer 2019). Schools already supported the biennial Cook Islands School Physical Health Examinations evaluating dental, skin, and hair health and physical development. The proposal of additional assessments associated with metabolic syndrome raised concerns. Education leaders acknowledged the potential value of these measures to inform evidence-based programme design but expressed apprehensions related to weight stigma, privacy, and lack of confidence that data could be meaningfully shared with participants (Wilson *et al*. 2023). The co-design process necessitates mutual agreement to progress planning. Health leaders respected the perspective of the education leaders, and consequently, a decision not to include metabolic health measures was reached.

By 2015, following the development and implementation of curriculum-linked learning exploring NCD risk and life course science (Bay 2017, Bay et al 2016a, b, 2019, Bay and Yaqona 2016), educators reported two significant observations: students wanted access to metabolic health measurements, and teachers wanted a better understanding of the metabolic health of adolescents. The team identified an opportunity to include some metabolic health indicators for Year 9 (Y9) students in an existing school-based health-monitoring programme. To mitigate risks, team members engaged in professional development and, with international colleagues, identified recommendations for integrating education and public health measures in school-based NCD risk reduction (Bay *et al*. 2017). These recommendations included timing to link the process to learning; resources to support students in accessing and understanding data; prioritising privacy; incorporating teacher and student feedback; and engaging in sense-making to promote the education, health, and community sectors to consider and respond to emerging evidence. Resource-associated factors limited the measurements to height, weight, waist circumference, blood pressure, blood glucose, and total cholesterol.

This significant undertaking encountered limitations associated with the SIDS context. Nevertheless, the *Y9 Metabolic Health Programme* is now integrated as a core component of Ora’anga Tūmanava. This study examined the development of the *Y9 Metabolic Health Programme* and analyses the steps that have supported its integration into education and public health practices in the Cook Islands. We examine the evidence that this programme has contributed to understanding of adolescent health in Rarotonga and present insights and future recommendations proposed by education and health professionals 8 years into the programme.

## MATERIALS AND METHODS

The design of PSHLP/Ora’anga Tūmanava integrates community-based participatory research (CBPR) with the Tivaevae research framework, reflecting and prioritising Kūki ʻĀirani (Cook Islands) epistemological and ontological worldviews (Futter-Puati and Maua-Hodges 2019, Wilson 2019). A Tivaevae is a traditional quilt, stitched as a gift for a special occasion. The design and stitching draw on the contributions of many, threading cultural knowledge and wisdom into the gift. Integrating CBPR and Tivaevae respects contributions from multiple knowledge systems in co-designing actions involving adolescents, families, education and public health professionals, and academics (Pineo *et al*. 2021). PSHLP emphasizes Kūki ʻĀirani leadership in engaging local, cultural, and traditional knowledge and wisdom in formulating actions to improve well-being and mitigate the health, social, and economic burdens that continue to affect peoples impacted by the legacy of colonisation (Chandanabhumma and Narasimhan 2019) . The process of stitching a Tivaevae engages the principals of tāʻokotaʻi (collaboration), ʻakairi kite (shared vision), tū ngāteitei (respect), tū ʻinangaro (relationships), and ʻuriʻuri kite (reciprocity) (Te Ava and Page 2020). Reflecting this cultural practice, decisions about programme development draw on skills, expertise, and experience from multiple people to metaphorically stitch research, learning, and actions. When a tivaevae is completed, it is viewed as a whole, forming a treasured gift that embodies wisdom and knowledge to be passed down generations. Futter-Puati and Maua-Hodges (2019) remind us that the intricacies of the stitching may not be seen when the tivaevae is viewed in its completed form, yet these hidden stitches have contributed to enabling its completion and beauty. In this study, we examine and document the layers of “patterns and stitching” that have contributed to the Y9 Metabolic Health Programme’s integration into learning and teaching and its potential to track metabolic health across generations in a manner that is sustainable for education and health and is acceptable within the community.

### Participants and consents

This study was conducted in Rarotonga, the main island of the Cook Islands; population 10 863 (Cook Islands Government 2022). Participating adolescents were enrolled in four schools, representing 96% of Y9 students in Rarotonga; the average annual Y9 cohort was 211. Te Marae Ora Cook Islands Ministry of Health gathered metabolic health data within routine health checks. Focus groups and interviews were used to collect feedback from students, teachers, and health staff at key points. Consent was confirmed by the heads of ministries (Education and Health), school principals, participating adults, and parents or caregivers. Adolescents aged under 16 years could provide assent once their parents or caregivers provided consent. Ethics approvals were obtained from The Cook Islands National Research Committee Ref. 05/14; Ref. 15/17; Ref 23/22; Ref 22/21; University of Auckland Human Participants Ethics Committee Ref. 011207; Ref. 23470; Ref. 22814; Auckland Health Research Ethics Committee Ref. 24668.

### Data collection

#### Health data

Health data collection occurred in school during class time via a series of collection stations staffed by health professionals, enabling assessment of the indicators listed in Table 1.

**Table 1.** Summary of health measures and indicators.

Measurement	Hardware/consumables	Process requirements	Indicator	Categories
Weight	SECA 813 digital scale	Barefoot, light clothing; to nearest 0.1 kg	Body Mass Index for Age (BMI-for-Age) (US Centres for Disease Control and Prevention 2024)	Underweight: < 5th percentile Healthy Weight: 5th to < 85th percentile Overweight: 85th to < 95th percentile Obesity: ≥ 95th percentile
Height	Portable, wall-mounted stadiometer	Barefoot; to nearest 0.1 cm		
Waist circumference	MyoTape flexible measuring tape	Midpoint between top of hip bone and bottom of ribs following exhalation; to nearest 0.1cm	Central Obesity Risk (New Zealand Ministry of Health 2020)	Low risk: WtHR ≤ 0.5 Increased risk: WtHR > 0.5
Blood pressure	Blood pressure cuff and Omron HEM-7203 automatic monitor	3–5 minutes seated resting; three measures, discard 1st measure	Blood Pressure (BP) (Flynn *et al*. 2017)	Normal: systolic BP (SBP) < 120 and diastolic BP (DBP) < 80 Elevated: SBP 120–129 and DBP < 80 High Normal/Stage 1 Hypertension (HTN 1): SBP 130–139 and/or DBP 80–89 High/Stage 2 Hypertension (HTN 2): SBP ≥ 140 and/or DBP ≥ 90
Blood glucose	Glucose strips, disposable lance, and SD Codefree glucose meter	Finger prick discarding 1st drop; record in mmol/l; fasting status recorded.	Blood Glucose (BG) (World Health Organization, n.d.)	*Fasting blood glucose* Normal: < 5.6mmol/l Raised: ≥ 5.6mmol/l *Random blood glucose* Normal: < 11.1 mmol/l Raised: ≥ 11.1mol/L
Total blood cholesterol	Cholesterol strips, disposable lance, Accutrend cholesterol meter	Finger prick discarding 1st drop; record in mmol/l; fasting status recorded	Total Cholesterol (U.S. Centers for Disease Control and Prevention n.d)	Normal: < 5.0 mmol/l Raised: ≥ 5.0 mmol/l

Measurements were recorded in spreadsheets and entered into each student’s personal Health Profile booklet (Herman et al. 2016). Follow-up protocols for metabolic measures outside the normal ranges involved contacting parents/caregivers within a week of the checks to discuss the results and enable appropriate referrals.

#### Participant perspectives

Data exploring participants’ perspectives were collected in 2016 via audio-recorded semi-structured small focus groups exploring students’ experiences within two weeks of the health check. Students who elected to participate could choose the size and composition of the focus group, a strategy used to support participant comfort. Focus groups were conducted by two researchers, one of whom was a Cook Islander. Conversations were conducted in English and could be interspersed with Kūki ʻĀirani reo Māori as is usual in the participating schools. Students were free to engage in conversations in Māori, with clarification requested if the Cook Islands researcher had questions.

#### Perspectives of health and education staff

In 2023/2024, focus groups and semi-structured interviews exploring the perspectives of education and health professionals regarding reflections on purpose, process, and outcomes were conducted using audio recordings or written responses. Following the protocols of the Ministries of Education and Health, these were conducted in English, often interspersed with Kūki ʻĀirani reo Māori. Participants were free to engage in Māori. All focus groups had at least one research team member who was fluent in both languages. As is common in workplace conversations in the Cook Islands, where interviews were conducted by a team member not fluent in Māori, participants who explored ideas in Māori followed up in English to support understanding for the non-Māori speaker.

### Data analysis

Within the Tivaevae model, data analysis engages collective decision-making, considering how evidence from different parts of the study stitch together to inform the research.

#### Quantitative data

Health data were analysed using Microsoft Excel (version 2503) to produce simple descriptive statistics before exploring differences between genders and collection years using significance testing with a 5% significance level (*P* < .05). Chi-square tests for BMI-for-age, waist-to-height ratio, total cholesterol, blood glucose, and blood pressure were used to compare categories for each measure against sex and study year as well as between any two measures. The Mann–Whitney U test for differences between sex for BMI-for-age (nonparametric alternative to the two-sample independent *t*-test) was additionally conducted due to BMI-for-age percentiles being typically non-normally distributed. For the same reason, the Kruskal–Wallis H test between study years for BMI-for-age (nonparametric alternative to a one-way ANOVA) was also conducted. There is a very small total island population and there are large differences in the rolls of the four schools. As expected, there were large differences in the number of students participating from each school. Analyses accounting for clustering at the school level were deemed unsuitable. This is due to the combination of small total island population, the nature of the population’s geospatial distribution and resultant high level of interactions, and school roll variability (as unequal cluster sizes can reduce statistical power). Analysis included linear (response variable of BMI-for-age percentiles, WtHR, fBSL, or cholesterol) or ordinal (response variable of BP categories) regression analysis using R Studio (version 4.3.1), with explanatory variables including an interaction term between study year and sex, and the remaining of the five measures.

This included chi-square tests for BMI, waist-to-height ratio, total cholesterol, blood glucose, and blood pressure, as well as the Mann–Whitney U test (for sex differences) and Kruskal–Wallis H test (for study year differences) for BMI-for-age.

#### Qualitative data

Audio files from focus group and interview data were transcribed manually (2016) or with approved AI assistance (2023/2024). Following transcription verification, qualitative data were reviewed for familiarisation. Utilising an iterative inductive approach, thematic analysis was conducted by teams, including Cook Islands members, to identify themes within the data (Kennedy 2018). The potential for researcher positionality to influence this process is recognised as significant. Consequently, collective sense-making discussions that acknowledged differences in positionality and relationality and prioritised perspectives from Cook Islands members were undertaken prior to theme confirmation. This recognised that in examining the data, researcher positionality and the relationship of the research team (collectively and individually) to the place and culture in which the work was occurring contributes to the conceptualisation of themes (Jadallah 2025). Assessment of trustworthiness of the process was guided by the criteria presented by Nowell *et al*.(2017), particularly the application of sense-making. In the context of this study, the utilisation of sense-making conversations between team members analysing data from diverse professional perspectives was also central to the assessment of trustworthiness. Where differences in perspectives were identified, further deliberation was undertaken to elucidate and confirm the themes.

## RESULTS

To examine the development and integration of the Y9 Metabolic Health Programme within PSHLP/Ora’anga Tūmanava, we present an overview of the development process alongside data relating to (i) student perspectives in 2016 that informed the process used from 2017 onwards, (ii) continuous improvement processes, (iii) metabolic health indicators 2016–2019 and 2022–2023, and (iv) programme review reflections from education and health staff in 2023/2024.

**Figure 1.**
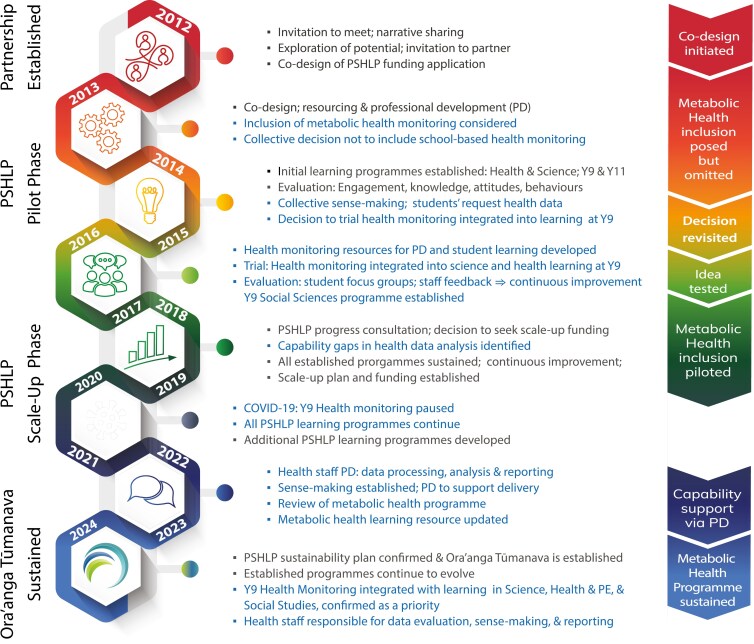
The evolution of metabolic health monitoring within the Pacific Science for Health Literacy Project. Black text indicates key events in the wider PSHLP initiative.

The development of the Y9 Metabolic Health Programme originated with respectful rejection of the proposal to include health measures in the PSHLP monitoring and evaluation plan in 2013. A shift in perspective occurred in the final quarter of 2015, during collective analysis of evidence from evaluations of the Y9 and Y11 learning programmes, which examined life course, social, and environmental factors associated with NCDs. Feedback revealed that students were seeking access to metabolic health indicators such as blood glucose, blood pressure, and cholesterol. In response to this feedback and considering educators’ concerns about weight stigma, privacy, and data access, a model was proposed that integrated metabolic health assessments into the existing Y9 learning programme, supported by specific resourcing and professional development (Herman *et al*. 2016). An overview of this programme’s evolution within PSHLP is outlined in Fig. 1.

### 2016: metabolic health pilot study year 1—student perspectives

**Figure 2.**
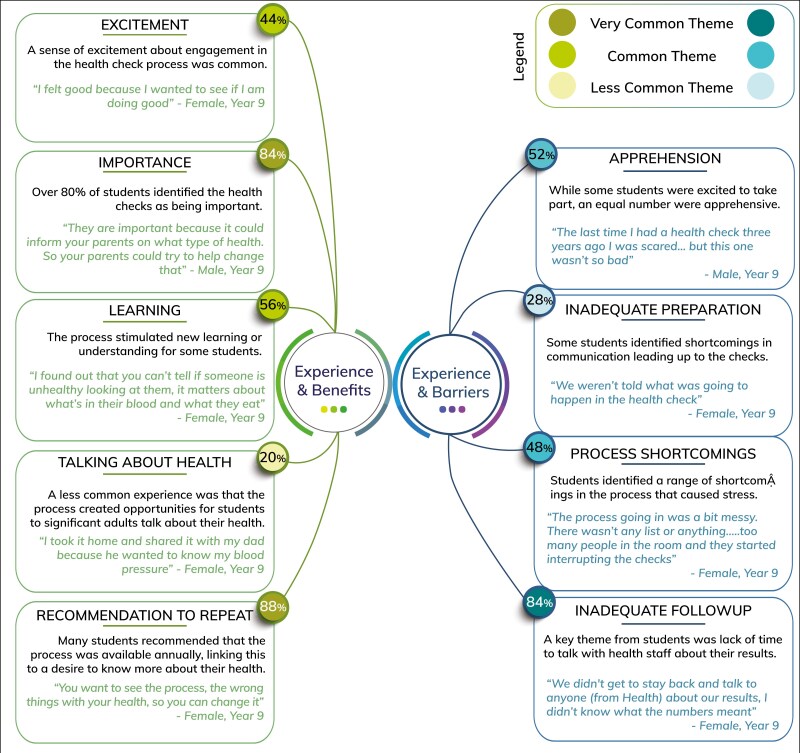
Student Voice 2016. Themes from focus groups with Year 9 students (*n* = 50) following participation in the initial metabolic health check process in 2016.

In the 2 weeks following the collection of metabolic health data from Y9 students in 2016, 15 mini-focus groups were conducted, involving 50 Y9 students (62% female) from two participating schools. Figure 2 provides a summary of themes emerging from these discussions.

Responses to the health check experience were evenly split between excitement and anxiety. Positive sentiments were linked to discovering health information, whereas negative sentiments stemmed from previous experiences and needle-related fears.

Despite their anxiety, most students identified the importance of health checks and deemed school an appropriate setting. Teacher feedback in 2015, identifying students’ interest in accessing and learning about their health data was reiterated in the focus groups. Students wanted to share health information with significant adults (typically parents or caregivers). Despite planning for all students to receive their data at testing, in 2016, 52% of students did not receive their data on the day, facing up to a 2 week wait. Students emphasised the importance of addressing the issue of waiting for data that some experienced and identified offering more time with health professionals to discuss the data as a priority.

Students overwhelmingly recommended that checks be offered annually, citing their ability to monitor their health and discuss it with parents or caregivers. Even students with negative experiences supported the programme’s continuation and suggested improvements. These included clearer precheck information on fasting and procedures as well as logistical enhancements for privacy and haste reduction.

### Continuous improvement

Student feedback in 2016 led to changes before, during, and after the checks. Since 2017, dedicated sessions on fasting and what to expect have been conducted before the checks. While achieved in most years, this has sometimes been limited by staffing constraints. In addition to newsletter communications, some schools use social media campaigns to remind students and parents about the checks and fasting.

Logistics during the checks were adjusted in 2017 and continue to be updated in response to feedback. Key points include reducing the number of people in the room, giving students more time to rest before blood pressure checks, and giving staff more time to talk with students. All checks are timetabled in the morning, and students are offered a light snack (fruit and cereal bars) following the event. All students receive results on the day, with check cards provided to any student who forgets to bring their booklet.

Some schools invite health staff to discuss data with students during the weeks following checks. Sense-making workshops in schools to enable teachers to discuss de-identified cumulative data with health staff were initiated in 2022.

The checks are timetabled to occur just before learning modules exploring NCDs, food environments, social change, and life course health (Barrett-Watson and Bay 2017, Bay and Yaqona 2016). Annual training sessions are held for health staff, supported by standard operating procedure documentation. Feedback from students regarding annual opportunities for checks has not been implemented due to capacity limitations. However, in 2023, the programme was extended to Year 10 for a 1-year trial, which we will report separately.

Education leaders who participated in examining the data in 2016 recommended the development of a sense-making programme to promote engagement with and understanding of the data for school staff, students, and families. This was delayed owing to capacity limitations and COVID-19. In 2022, professional development was introduced to boost the confidence of health staff in analysing and communicating data. Templates were developed to assist in the production of data summaries for use in school communities, enabling sense-making workshops to be conducted in participating schools. By 2023, health staff fully managed data analysis, with statistical review supported by the partner university. To address challenges associated with high staff turnover in a small population, online tools were developed to aid professional learning and ensure the continuity of data analysis and sense-making processes (Pacific Science for Health Literacy Project Team 2024). Learning resources that facilitate exploration of NCDs locally and globally have been updated to include population-level summaries of adolescent metabolic risk, supporting teachers to engage students in conversations about the prevalence and complexity of risk factors.

### Metabolic health indicators (2016–2023)

From 2016 to 2019 and 2022 to 2023, data were collected from 783 Y9 students, representing 67.6% of Y9 students in the 4 participating schools and 64.5% of the total Y9 population for the island of Rarotonga. Participation reflected student distribution across the schools; gender was balanced (51.9% female); median age 13.8 years (Table 2). On average, ~85% of school students in Rarotonga are of Cook Islands Māori descent. There was no significant difference in the distribution of sex across years compared with what would be expected if independent (χ^2^  *P* = .306).

**Table 2.** Participation by study year and school.

		Participants by school (*n*)							Participation rate	
Year		A	B	C	D	Total	Median age (years)	% Female	% Y9 cohort in participating schools	% Total Y9 cohort in Rarotonga
2016	Yr 9	50	13	NA	7	70	13.8	54.3	42.4	38.5
2017	Yr 9	117	22	NA	NA	139	13.8	51.1	83.2	73.9
2018	Yr 9	84	17	10	9	120	14.1	52.5	67.0	64.9
2019	Yr 9	93	32	NA	11	136	13.7	51.5	65.7	63.6
2022	Yr 9	128	24	7	11	170	13.8	45.3	81.7	75.2
2023	Yr 9	100	26	4	18	148	13.6	58.8	72.5	67.6
Total		572	134	21	56	783	13.8	51.9	67.6	64.5

**Figure 3.**
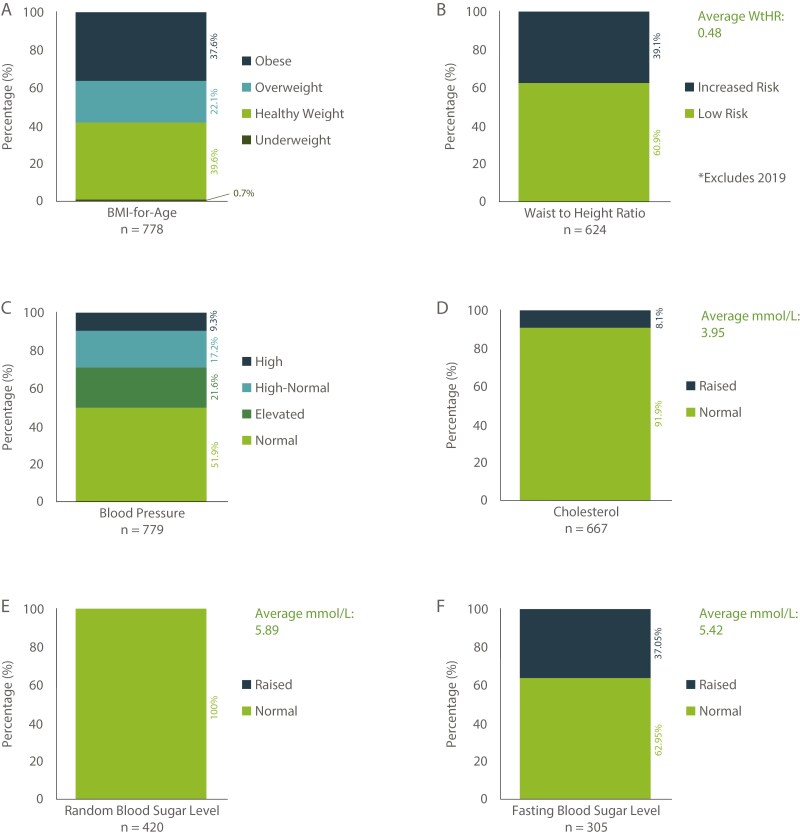
Cumulative Y9 cohort 2016–2023, percentage distribution for (a) BMI-for-Age, (b) WtHR, (c) BP, (d) Cholesterol, (e) Random BSL, and (f) Fasting BSL.

Figure 3 presents data for the combined Y9 cohorts (*n* = 783) showing percentage distribution of Body-Mass-Index-for-Age (BMI-for-Age, kg/m^2^), Waist-to-Height Ratio (WtHR), Blood Pressure (BP, mmHg), Blood Cholesterol (mmol/l), and Random and Fasting Blood Sugar Levels (BSL, mmol/l). Risk factors observed included 22.1% overweight, 37.5% obese, 39.1% with increased WtHR, 8.1% with elevated blood cholesterol, 24.0% with elevated BSL, and 9.4% with high BP.

There was no significant difference in the distribution of BMI-for-Age categories across years (χ^2^  *P* = .19, Kruskal–Wallis H test *P* = .609, ANOVA *P* = .959). There was no significant difference between sexes for BMI-for-age (χ^2^  *P* = .233, Mann–Whitney U test *P* = .170, *t*-test *P* = .881). There was no significant effect of study year, sex, or their interaction on BMI-for-age percentiles (linear regression, *P* = .89), indicating that BMI percentiles remained consistent across years and between sexes.

All other variables showed a significant difference across years (WtHR χ^2^  *P* < .001; BP χ^2^  *P* = .005; cholesterol χ^2^  *P* = .003; fBSL χ^2^  *P* = .007) and an ANOVA (WtHR *P* < .001; cholesterol *P* < .001; fBSL *P* = .011). Observations in 2018 were a key contributor to significant *P*-values. There was no significant difference between sexes for all variables (WtHR χ^2^  *P* = .194; cholesterol χ^2^  *P* = .703; fBSL χ^2^  *P* = .210) except BP (χ^2^  *P* < .001). Specifically, female normal BP levels were higher than expected, while male normal BP levels were lower than expected. A set of two-sample *t*-tests assuming unequal variances found much the same for WtHR and fBSL (WtHR *P* = .198; fBSL *P* = .114), although the *t*-test for cholesterol was significantly different between sex (*P* = .040, average cholesterol for males = 3.85 compared with females = 3.96) suggesting a difference in average cholesterol levels but not a difference in proportion of at-risk between sexes. Utilising linear regression, there was a significant effect of study year (but not sex or the year–sex interaction) on WtHR (linear regression, 2018, *P* = .007). There was a modest but statistically significant effect of year and sex on fBSL (*P* = .033), though individual year-to-year or sex-based differences were not statistically significant. By comparison, cholesterol levels varied significantly across study years (*P* < .001), with significant increases observed in 2017–2023 relative to the 2016 baseline, and a significant negative interaction in 2017 indicating lower cholesterol among males compared with females that year. A cumulative link model indicated that, on average, males had higher odds than females of being in a higher BP category overall (*P* = .004), with significant negative interaction effects in 2017 and 2019 indicating lower BP odds for males in those years compared with males in 2016 (*P* = .035 and *P* = .040, respectively).

Five risk factors related to metabolic syndrome were measured: BMI-for-Age in or above the overweight category, WtHR > 0.5, BP at or above high normal (excluding elevated BP due to the potential of stress related to data collection), raised BSL (fasting or random), and raised total blood cholesterol levels (DeBoer 2019). Data for all five measures were available for 321 participants, representing 41% of the sample. In this subsample, 27.7% were living with three or more risk factors. A significant difference between females and males was identified (χ^2^  *P* = .041). Fewer females and more males with three or more risk factors were observed than expected.

Other variable comparisons indicated higher than normal BP was significantly associated with BMI-for-Age categories (*P* < .001), WtHR (*P* < .001), and cholesterol (*P* < .001). There were no other significant interactions between variables. Linear regression models with study year–sex interaction effects indicated significant relationships between BMI-for-age percentile and WtHR (*P* < .001), BMI-for-age percentile and normal BP (*P* < .001), and fBSL and normal BP (*P* < .047).

### Reflections from education and public health professionals (2023/24)

Annual planning and ongoing improvements are informed by feedback from education and health professionals, including teachers’ insights into student learning. We present feedback collected from six educators and four public health staff via group discussions and individual conversations in 2023/2024. Five participants had been involved since the programme’s inception in 2015; the other five had engaged for at least 3 years. All but one were Cook Islanders or permanent residents.

Programme benefits were identified as impacting participants, as well as the work of the Ministries of Education and Health. At the level of participants, the opportunity to access and understand health data was identified as a benefit associated with agency and action.

“Students to get a snapshot of metabolic health indicators. They can use their data in decision-making around lifestyle choices. Learning gives them knowledge of the [related] science and social science… The testing and learning give the students a sense of ownership of their health and an understanding of the why. Many have shared in class that they have shared knowledge with their families particularly those who have NCDs as they are concerned about them.” Educator A

“Some parents were talking about the health profile. They really appreciated that the students got that opportunity to take it [the health profile booklet] back home, they had a look at it.” Educator D

Education and health staff reported mainly positive engagement from students, acknowledging that some approach the health measures with caution. Teachers emphasised the impact of associated learning experiences exploring aspects of the NCD challenge across science, health and PE, and social studies in promoting positive engagement with the health measures. Simple strategies, such as offering students a chance to talk privately or return later, have been found to be effective for supporting anxious students.

“There is an obvious increase in students wanting to learn more about and manage their own health and wellbeing and the health checks provide a great starting point for this.” Educator C

“…. when we go back and revisit this [health checks], they say, oh, mister, my blood sugar level is nearly seven…. they were sharing …and they discuss it.” Educator B

“Most of the students were agreeable to the health checks and look forward to knowing their results… Some were curious…. Some were hesitant …they even raised the question of, why me and why now? A few didn’t want to [take part] …maybe shy… maybe scared of the unknown.” Health Staff F

Within the education sector, the programme is seen as supporting holistic approaches to learning and teaching and informing education policy.

“The health checks offered the Ministry [of Education] actionable data to understand the health status of our learners. It has enhanced schools’ capacity to integrate health literacy across the curriculum, equipping teachers with confidence and resources to address health topics and issues. This initiative strengthens the alignment of education and health priorities, emphasising the importance of a whole-child/ whole-family/whole-community approach to learning.” Educator C

It was also noted that the benefits of increased awareness of metabolic health may extend beyond the professional setting to the personal lives of staff.

A benefit is also “raised awareness across school staff of NCDs and issues they or their loved ones may be facing.” Educator A

Within the health sector, the programme is similarly identified as offering evidence to support NCD risk-related policy and planning. Professional development associated with structured, scaffolded support for data analysis, reporting, and communication is identified as a significant benefit, addressing capability gaps identified by staff.

“The programme has enhanced our capacity to analyse and present the health check data. Access to tools (SPSS) and training like workshops and one-on-one mentoring has supported my team to move from learning and co-analysis to being able to take over the analysis, with the confidence that our team can do this internally. We still have access to support if we need it.” Health Staff J

The Y9 metabolic health check programme has been embedded into core business in both sectors. Feedback indicates that efforts to build this partnership have supported the programme to date, and the future commitment including growth of the programme to include a wider age range.

“Working collaboratively with Ministry of Education and the schools strengthens the capacity to deliver health services in the schools. Data gained from the metabolic health check will guide evidence based policymaking and future education initiatives.” Health Staff F

“The Ministry of Health has used schools as a site for health examinations for many years. The work we have undertaken to develop the metabolic health checks has changed the way this happens. Now the health teams can go in and do school health checks with a lot more wraparound support for the students and their learning – that has come about because of Ora’anga Tūmanava.” Educator E

All partners acknowledge ongoing challenges associated with cross-sectoral partnerships in the context of a small island state. These particularly relate to fluctuating staffing capacity and reliance on short-term expatriate staff, particularly in the education sector. This increases the importance of ongoing investment in building and maintaining the partnership. Strategies have been developed to formalise this commitment, including embedding professional development resources aimed at introducing the programme to new staff and continuously refreshing awareness and capabilities of existing staff.

Safeguarding sensitive data and ensuring that staff possessed the necessary data-handling skills were identified as concerns in 2015. Staff confirmed that these risks were being effectively addressed.

“Ensuring student health data is handled confidently, and teachers and health professionals have the best level of training to deliver the health checks effectively could be considered risks... however, we know these are consistently being addressed and managed.” Educator C

Looking ahead, respondents recommended future actions to enhance the health check programme. These included expanding health checks to other year levels to integrate with the development of health literacy throughout a student’s learning journey. They emphasised the importance of sustained partnerships between health and education to ensure continued support and noted the value of this programme to the recently formed Cook Islands NCD Task Force. This multi-agency group is tasked with coordinating efforts towards NCD risk reduction. Additionally, they suggested that long-term tracking of the impact of the health checks on learners’ academic and health outcomes would be a fruitful area for research.

## DISCUSSION

This study explored factors that transformed the initial concept of metabolic health tracking, proposed but not adopted in 2013, into a priority programme addressing an evidence gap in adolescent health data, providing teenagers with meaningful data access and strongly endorsed by schools and health.

Longitudinal monitoring of child and adolescent health informs individual- and population-level interventions for current and future generations (Patton *et al*. 2016, World Health Organization 2024). In the Cook Islands, the primary method for monitoring child and adolescent health on a population scale involves the biennial School Physical Health Examinations for students aged 5–18 years. This provides valuable evidence, including BMI-for-age, but does not assess additional factors associated with metabolic syndrome. By attending to perspectives from education and health, the Y9 Metabolic Health Programme has generated valid health evidence in a manner that is acceptable to schools and respectful of students’ rights to access and understand their health data.

We identified four integrated factors that contributed to establishing this societally acceptable methodology to monitor and enhance the understanding of metabolic health risk in adolescents in Rarotonga. These encompass trust, safety, data access and comprehension, and addressing capability and capacity challenges in the context of a small island state in the South Pacific. The processes followed in the development of the programme drew on the principles of the Tivaevae model: tāʻokotaʻi (collaboration), ʻakairi kite (shared vision), tū ngāteitei (respect), tū ʻinangaro (relationships), and ʻuriʻuri kite (reciprocity) (Te Ava and Page 2020). These principles reflect the importance of Pacific knowledges and values in developing research that embodies Pacific aspirations, is contextually responsive, transdisciplinary in nature, and can offer meaningful contributions in Pacific societies (Anae *et al*. 2001).

The importance of respect, trust, and willingness to wait for consensus before acting is reflected in the co-design journey. In the 2013 planning, the health sector respected the concerns of educators regarding health measurements in schools. This enabled learning and teaching programmes exploring the complexity of NCDs via science, social science, and health to develop without conflict. These programmes promoted increased engagement with and understanding of NCD-related issues across the education sector, and within 2 years, the need for engaging in longitudinal measures of metabolic health indicators during adolescence became a desired component of PSHLP. This demonstrates the long-term benefit of working toward consensus to enable a vision that respects the perspectives of all partners. This concept of shared vision and co-design of strategy considering health and education perspectives was identified by the team in their analysis of the challenges and opportunities of school-based primary NCD risk reduction undertaken in 2016 (Bay *et al*. 2017). This is similarly identified as the core foundation for successful partnerships associated with sustainable development in broader settings (Stott and Murphy 2020, Leal Filho *et al*. 2024). Our work demonstrates the potential created by integrating diverse perspectives in co-design and implementation for sustainable actions to address complex issues. We acknowledge that our small island state context, while often associated with challenges, offers the benefit of tight connections between government agencies and communities that likely enhance the potential for this approach to be successful.

Safety concerns expressed by educators in 2013 related to managing issues of weight stigma, privacy, and confidence that data could be meaningfully shared with participants. By 2015, trusting relationships, reciprocity, and shared vision had strengthened within the team. Educators had greater understanding of the health team’s goal to track metabolic risk indicators and identified how, if undertaken with care, this could strengthen education outcomes. The decision to link data into curriculum and learning illustrates a shift in perspectives regarding the purpose and use of health evidence. This repositions the use of metabolic indicators previously focusing primarily on what health professionals might know about the health status of young people. It improves the value proposition of the collection of health data in schools by increasing ownership of actionable knowledge relevant to teachers, adolescents, and families (Fors and Pink 2017). Significant insights from the programme’s development relate to how collaborators have addressed the complexities surrounding the appropriate involvement of schools as a setting for population-level health monitoring, ensuring safety, access to evidence, and connections to educational goals to promote understanding. Previous work has focused on data-sharing agreements between health and education, the impact of health disparities on academic performance, and the effective use of health data in schools to close achievement gaps and promote overall wellbeing (Basch 2011, Ickovics *et al*. 2014). The Y9 Metabolic Health Programme has emphasised the importance of access to and understanding of personal data for participating adolescents. Future research could explore how this engagement impacts health-related decision-making for schools and adolescents.

Confidence in the quality and validity of the data is a further factor associated with safety and trust and impacted by capability and capacity within the system. Professional development alongside regular cycles of reflection and continuous improvement contributed to addressing these concerns.

Effective public health strategies depend on reliable information, which is often lacking for smaller populations (Korngiebel *et al*. 2015). Population size is an ongoing challenge related to the importance of local evidence in small-island settings. In addition to being valuable to health planning, local evidence is a priority for education planning and is known to enhance student engagement in learning (Hunter and Hunter 2024). As an efficient, low-cost approach, a repeated cross-sectional design was used in this study, providing generalisable insights into changes in health outcomes over time (Wang and Cheng 2020).

This design addressed concerns about small population size and over a period of several years, enabled valid evidence to emerge. Heightened risk factors associated with metabolic syndrome are evident in the population of Y9 students sampled from 2016 to 2023. This is unsurprising given the levels of metabolic syndrome risk in the adult population (Te Marae Ora Ministry of Health Cook Islands 2022). The findings align with evidence from other Pacific Island populations (Kessaram *et al*. 2015, Plank *et al*. 2019, Phelps *et al*. 2024) and demonstrate the contribution of the programme to local evidence. The BMI-for-Age data (59.7% ≥ 85th percentile; 37.6% ≥ 95th percentile) are similar to the self-reported data from the 2015 WHO Global School Based Health Survey for students in the Cook Islands aged 13–15 years (*n* = 363) (58% ≥ 85th percentile; 31% ≥ 95th percentile) (Te Marae Ora Ministry of Health Cook Islands 2023). However, our data are measured rather than self-reported and, importantly, offer a greater depth of understanding of the development of risk factors associated with metabolic syndrome. Our findings reveal that BMI-for-Age and WtHR were good indicators of a participant living with three or more risk factors. Population-level evidence of emerging risks associated with metabolic syndrome in children and adolescents could be strengthened by adding waist circumference measures into the biennial Cook Islands School Physical Health Examination programme. This would enable the combination of BMI and WtHR to be utilised to track risk across the 5- to 18-year-old population, including in the remote outer islands.

Tivaevae draws on knowledge and wisdom of many to stitch patterns and symbols into a gift that tells a story that can be used now and by generations to come. The richness of the story of this research is found in multiple layers of stitching that draw on knowledge from health, science, education, and culture. Our research gifts new evidence about the health of adolescents to participants, their families, and the community. By participating, young people know more about their health and the health of their community. By viewing the research, families, the community, and government can see why it is so important to continue to prioritise the health of young people. This new evidence can be used to track the impacts of system-level changes on adolescent health, and the programme can be used to continue to build evidence. The research findings are made possible by the often-unseen layers of interactions between people, values, and knowledge that enabled the programme to evolve. Relationship building, professional development, sense-making, learning, sharing, and collaboration are stitched into the base of the tivaevae, providing the platform through which the data for health, education, young people, and their families can be made visible. Community-based participatory research draws on rich interactions between multiple knowledge systems. This research demonstrates how local leadership and ownership that promotes tāʻokotaʻi (collaboration), ʻakairi kite (shared vision), tū ngāteitei (respect), tū ʻinangaro (relationships), and ʻuriʻuri kite (reciprocity) can weave together the strengths of education, health, and community to drive health promotion.

## LIMITATIONS AND FUTURE RESEARCH

Potential limitations are acknowledged to provide context for interpreting findings and identifying areas for development.

Student input via mini-focus groups was formalised in 2016 to contribute to continuous improvement. In subsequent years, teachers, education leaders, and health staff contributed feedback on student engagement and responses at annual planning meetings, which drove the continuous improvement cycle. A limitation of this study was the use of secondary data regarding students’ perspectives, with the exception of 2016. The primary data collection related to perspectives in 2023/2024 were limited to education and health staff owing to resourcing. Repeating the exploration of programme experience with students periodically would be beneficial.

The 2018 data presented unexplained variability. This inconsistency may reflect measurement errors, as data collection involved numerous individuals across years, particularly considering that repeated measurements using the same method can naturally yield slight variations (Brakenhoff 2018). This variability strengthens the rationale for continued professional learning to support adherence to established and co-developed standard operating procedures. This limitation also reinforces the value of a repeated cross-sectional design in identifying trends and maintaining consistency over time.

The Pa Enua (outer islands) have engaged in the associated science, social studies, and health programmes but are only just starting to integrate the metabolic health checks into the Healthy Islands Health Promotion Programme. Future research may usefully compare findings across the diverse settings of different Pa Enua and Rarotonga.

Sense-making workshops have been discussed within annual continuous improvement cycles but are yet to be formally evaluated. Exploration of how schools are using the data to inform learning, teaching, and pastoral care would be of value.

## CONCLUSIONS

The NCD burden on the Cook Islands is extreme. This research provides increased evidence of the prevalence of risks associated with metabolic syndrome in the early teenage years, highlighting the importance of investing in child and adolescent health to reduce non-communicable disease burdens. We reported on lessons learned in developing a health promotion programme that co-creates actionable knowledge relevant to adolescents, their families, and the education and health sectors within the constraints of a small population in a limited resource setting. Trust between health and education is a significant factor in enabling schools as a setting for health promotion. The evolution of the Y9 Metabolic Health Programme in Rarotonga represents a significant investment of time that strengthened tū ʻinangaro (relationships) and ʻuriʻuri kite (reciprocity) across education and health in the Cook Islands. Within transdisciplinary partnerships, recognising how each partner’s sector-specific objectives align with the overarching shared goal unlocks the full potential of a unified vision. The process of understanding why the education sector was concerned about the use of schools as a setting for health measures led to conversations that supported partners to better understand sector-specific needs and perspectives. Listening to feedback from teenagers revealed actions that supported their engagement. Enhancing awareness of health issues alongside addressing capability gaps, safety, and data access and comprehension were identified as crucial areas for development. Collectively, these actions supported the evolution of the Y9 Metabolic Health Programme from a pilot initiative in 2016 to a sustained, integrated programme contributing within the holistic strategy for NCD risk reduction in the Cook Islands.

## Supplementary Material

daaf069_suppl_Supplementary

## Data Availability

The data presented in this article cannot be shared publicly due to ethical requirements. Detailed summary tables are provided in the Supplementary File.
