# Supplementary material for: Evolution of a sustained health promotion programme exploring adolescent metabolic health in the Cook Islands
Source: Health Promot Int. 2025 Jun 26;40(3):daaf069. doi: 10.1093/heapro/daaf069 (PMC12199598; doi:10.1093/heapro/daaf069)
Supplement: daaf069_suppl_Supplementary [file daaf069_suppl_supplementary.docx]

Table A. Variable average measures and standard deviation by year of study and sex.

|  | **Average ± SD** | | | | | |
| --- | --- | --- | --- | --- | --- | --- |
| **Year** | **BMI-for-Age (kg/m^2^)** | **Waist to Height Ratio** | **Cholesterol (mmol/L)** | **Heart Rate (bpm)** | **Fasting BSL (mmol/L)** | **Random BSL (mmol/L)** |
| **2016** | 24.6 ± 5.4 | 0.47 ± 0.09 | 3.51 ± 0.71 | - | 5.18 ± 0.04 | 5.72 ± 0.80 |
| **2017** | 24.8 ± 5.5 | 0.50 ± 0.08 | 3.62 ± 0.83 | 87.1 ± 16.1 | 5.59 ± 0.19 | 5.88 ± 0.72 |
| **2018** | 25.2 ± 6.2 | 0.52 ± 0.07 | 4.15 ± 0.44 | 79.3 ± 14.5 | 5.15 ± 0.18 | 5.49 ± 0.69 |
| **2019** | 24.8 ± 5.7 | - | 4.29 ± 0.50 | - | 5.54 ± 0.68 | 6.45 ± 1.01 |
| **2022** | 24.6 ± 6.1 | 0.47 ± 0.08 | 4.27 ± 0.44 | 85.2 ± 14.9 | 5.61 ± 0.55 | 6.05 ± 0.80 |
| **2023** | 25.0 ± 5.9 | 0.38 ± 0.16 | 3.76 ± 0.80 | - | 5.41 ± 0.42 | - |
| **Total** | **24.9 ± 5.8** | **0.45 ± 0.12** | **3.91 ± 0.73** | **84.2 ± 15.5** | **5.44 ± 0.65** | **5.89 ± 0.84** |
| **Females** | **24.9 ± 5.5** | **0.45 ± 0.12** | **3.96 ± 0.72** | **86.1 ± 15.2** | **5.39 ± 0.62** | **5.81 ± 0.84** |
| **Males** | **24.8 ± 6.2** | **0.46 ± 0.13** | **3.85 ± 0.74** | **82.3 ± 15.5** | **5.49 ± 0.67** | **5.98 ± 0.83** |
